# Computed tomographic assessment of airflow obstruction in smoke inhalation injury

**DOI:** 10.1186/cc13447

**Published:** 2014-03-17

**Authors:** H Yamamura, T Yamamoto, S Kaga, K Kaneda, Y Mizobata

**Affiliations:** 1 Osaka City University, Osaka, Japan

## Introduction

Smoke inhalation injury (SII) is progress to pulmonary edema, pneumonia, and acute respiratory distress syndrome. SII may cause bronchial mucosal edema; we hypothesized that narrowing of luminal air bronchus due to bronchial wall edema correlated with respiratory deterioration of SII patients.

## Methods

We prospectively studied 42 patients with a diagnosis of SII, according to visualized bronchoscopic findings at admission, and 15 control subjects. The thoracic high-resolution computed tomography (HRCT) scan was obtained within a few hours of admission to our hospital. Airway wall dimensions were calculated using a validated method. The images were viewed on a workstation using a magnification of ×5, and measurements of overall (D) and internal (L) diameter of the bronchi were made using electronic calipers, with wall thickness (T) being derived from these measurements (T = (D - L) / 2). Luminal area (Ai, mm^2^) and total airway wall area (Ao) were calculated from L and D, respectively, using the formula: A = πr^2^. We used both the ratio of airway wall thickness to total diameter (T/D ratio) and the percentage luminal area (LA% = (Ai / Ao + Ai) × 100).

## Results

The mean age of the patients was 59 years, 32 of the patients were men. The mean (SD) diameter of the bronchi in SII patients measured was 3.9 (1.5) mm (range 0.9 to 9.0 mm). There were statistically significant positive associations between wall thickening (expressed as T/D ratio) and luminal narrow (expressed as LA%) and the developed pneumonia (T/D ratio: *r*^2 ^= 0.56, *P *< 0.01 and LA%: *r*^2 ^= 0.19, *P *= 0.005) and mechanical ventilation days (T/D ratio: *r*^2 ^= 0.37, *P *< 0.0001 and LA%: *r*^2 ^= 0.32, *P *< 0.001, respectively). No statistically significant associations were identified between T/D ratio or LA% and initial P/F ratio, infusion volume initial 24 hours, ICU stay days, and outcome. The mean T/D ratio and LA% were 0.25 (0.04) and 25.9% (7.6) for patients with SII and 0.35 (0.04) and 44.7% (5.6) for controls.

## Conclusion

We have shown with the use of HRCT scanning on admission that patients with SII have airway wall thickening compared with normal controls. Furthermore, airflow obstruction due to bronchial wall edema related with developed pneumonia and mechanical ventilation days in SII patients.

